# Plant-based proteins: clinical and technological importance

**DOI:** 10.1007/s10068-024-01600-5

**Published:** 2024-07-02

**Authors:** Isabel Medina-Vera, Azalia Avila-Nava, Liliana León-López, Ana Ligia Gutiérrez-Solis, José Moisés Talamantes-Gómez, Claudia C. Márquez-Mota

**Affiliations:** 1 Departamento de Metodología de la Investigación, Instituto Nacional de Pediatría (INP), Mexico City, Mexico; 2 Hospital Regional de Alta Especialidad de la Península de Yucatán, Mérida, Mexico; 3 Programa de Posgrado Integral en Biotecnología, Facultad de Ciencias Químico-Biológicas, Universidad Autónoma de Sinaloa, C.P. 80000 Culiacán, Sinaloa Mexico; 4 Departamento de Nutrición Animal y Bioquímica, Facultad de Medicina Veterinaria y Zootecnia (FMVZ), Universidad Nacional Autónoma de México, Mexico City, Mexico

**Keywords:** Plant-based protein, Bioactive peptides, Protein signaling pathways, Muscle synthesis

## Abstract

Healthy and sustainable diets have seen a surge in popularity in recent years, driven by a desire to consume foods that not only help health but also have a favorable influence on the environment, such as plant-based proteins. This has created controversy because plant-based proteins may not always contain all the amino acids required by the organism. However, protein extraction methods have been developed due to technological advancements to boost their nutritional worth. Furthermore, certain chemicals, such as bioactive peptides, have been identified and linked to favorable health effects. As a result, the current analysis focuses on the primary plant-based protein sources, their chemical composition, and the molecular mechanism activated by the amino acid types of present. It also discusses plant protein extraction techniques, bioactive substances derived from these sources, product development using plant protein, and the therapeutic benefits of these plant-based proteins in clinical research.

## Introduction

### Dietary protein

Proteins are composed of amino acids held together by peptide bonds. Dietary proteins are essential for maintaining physiological equilibrium; hence, they are a vital component of the diet derived from animal or plant sources. However, debate exists since the quality of the protein varies depending on its source. Several factors, including the types of amino acids, concentration, and digestibility, influence the nutritional value of the protein (Hendriks et al., 2012).

The hydrolysis of proteins into amino acids, dipeptides, and tripeptides in the lumen of the small intestine determines their digestibility. Amino acids are components that supply the organism with nitrogen, hydrocarbon skeletons, and sulfur. There are twenty amino acids, and nine are considered essential amino acids (EAA), meaning they cannot be synthesized by the body and must be obtained from exogenous sources such as the diet. Leucine, valine, isoleucine, histidine, lysine, methionine, threonine, tryptophan, and phenylalanine are the EAA. Since amino acids are precursors for the synthesis of biomolecules such as proteins, peptides, and low-molecular-weight substances, their physiological significance influences a variety of physiological and metabolic processes (Wu, 2016).

Due to the significance of protein quantity and nutritional value based on amino acid content, various methods of reporting these characteristics have been described and utilized. Since most amino acids contain 16% nitrogen, the amount of protein is typically reported as the total nitrogen content multiplied by 6.25. However, this does not account for other nitrogenous compounds; therefore, the protein content may be under- or over-represented (Watford and Wu, 2018).

On the other hand, many methods have been created to assess their nutritional value according to their digestibility. The most common method was based on the Protein Efficiency Ratio (PER) (determined in growing rats) for many years. Currently, other methods have been implemented, such as Protein Digestibility-Corrected Amino Acid Score (PDCAAS) and Digestible Indispensable Amino Acid Score (DIAAS). However, the values obtained in PDCAAS may overestimate protein quality, as it considers protein digestibility through fecal analysis, which does not consider that the disappearance of nitrogen in the large intestine is not due to protein digestion and absorption, but to microbial degradation, which results in ammonia production, absorption, and excretion as urine (Hendriks et al., 2012). In this sense, DIAAS is more specific as it considers ileal digestibility, and values are not truncated at 1.0, as they are with the PDCAAS (Nichele et al., 2022) (Table 1).

**Table 1:** Protein digestibility evaluation methods

Digestibility method	Fundament	Formula	Limitation	References
PER	Ratio of weight gain by intake of target protein over reference protein	$\frac{{{\text{Weight gain (g) TP/ Intake of TP (g)}}}}{{{\text{Weight gain (g) RP/ Intake of RP (g)}}}}$	Underestimate the quality of some vegetable proteins	Consultation (2011) and Schaafsma (2012)
PDCAAS	Ratio of indispensable amino acids in target protein over reference protein corrected by protein digestibility	$\frac{{{\text{IAA}}_{{\lim }} {\text{ TP }}({\text{mg/g TP}})}}{{{\text{IAA}}_{{\lim }} {\text{ RP }}({\text{mg/g RP}})}} \times {\text{Fecal}}\,{\text{digestibility}}\,{\text{TP}}\%$	Overestimate the protein quality	Consultation (2011)
DIAAS	Ratio of indispensable amino acids in target protein over reference protein corrected for digestibility of IAA_lim_	$\frac{{{\text{IAA}}_{{\lim }} {\text{ TP }}({\text{mg/g TP}})}}{{{\text{IAA}}_{{\lim }} {\text{ RP }}({\text{mg/g RP}})}} \times {\text{Ileal}}\,{\text{digestibility}}\,{\text{IAA}}_{{\lim }} \%$	Underestimate the quality of some vegetable proteins	Consultation (2011)

*PER* Protein efficiency ratio, *TP* target protein, *RP* reference protein, *PDCAAS* protein digestibility-corrected amino acid score, *IAA*_*lim*_ indispensable limiting amino acid, *DIAAS* digestible indispensable amino acid score

Despite these protein quality assessments, current recommendations regarding the amount of protein intake do not directly address the quality of the protein. It is also important to consider the Acceptable Macronutrient Distribution Range (AMDR), which considers a range of protein intake providing between 10 and 35% of daily calories in the diet, and the Recommended Dietary Allowance (RDA) for protein is 0.80 g of “good quality protein” per kg of body weight per day (g/kg/day) (Trumbo et al., 2002). It considers that the quality of the protein should not only depend on this characteristic but also on other health-related benefits. Therefore, the protein quality must take into consideration the “whole food package,” or the “protein package,” which refers to the other components present in foods used as sources of protein (Mariotti, 2019).

### Importance of plant-based protein

The selection and type of food in the diet have direct effects on the environment and the health of the population. Only a few foods contain large amounts of protein; in economically developed countries, the usual intake is between 12 and 20% of total energy intake. This is because the foods with the highest amount of protein are of animal origin, so there is a debate about the amounts of fats and carbohydrates contained in these foods and the role of their long-term consumption on health issues (Mariotti, 2019).

In 2010, the Food and Agriculture Organization (FAO) defined sustainable diets as “diets with low environmental impacts which contribute to food and nutrition security and to healthy life for present and future generation”. This considered other economic, social, and environmental factors that protect and respect biodiversity and ecosystems, are culturally acceptable, accessible, economically fair, and affordable (Burlingame and Dernini, 2010). In this sense, plant protein production generally requires fewer natural and economic resources, including land, water, and energy, than obtaining animal protein. Thus, a plant-based diet is the most effective strategy for systemically reducing consumption-accounted greenhouse gas emissions (GHGEs) and agricultural land use related to food production and consumption (Lynch et al., 2018).

Currently, the consumption of plant-based diets plays an important role in the presence of bioactive components, such as vitamins, polyphenols, or bioactive peptides. Hence, these components benefit human health and protect against various disease conditions (Langyan et al., 2022). There is evidence that plant-based protein foods contributed more to the intake of nutrients such as dietary fiber, vitamin E, magnesium, and polyunsaturated fatty acids (PUFA) compared with animal-based protein that included the intake of cholesterol and saturated fatty acids, which promoted a nutritionally adequate, safe, and healthy diet while optimizing natural and human resources.

### Sources of plant-based protein

There are different sources of vegetable proteins, which vary in quantity and types of protein, as well as in the variety of amino acids that compose them, and some of these vegetable foods with protein contributions include oats, corn, soybeans, amaranth, peanuts, and walnuts (Table 2).

**Table 2:** Characteristics of the source of plant-based protein

Source	Protein content (%)	Types of protein	Amino acids	References
Oats	11–24.5	Globulin	Source of glutamic acid and leucine	Mäkinen et al. (2017)
Corn	7–13	Globulin, albumin, glutelin, and zein	Limited content of tryptophan and lysine	Joshi et al. (2022)
Soybean	35–40	Globulins	Source of all essential amino acids: histidine, isoleucine, leucine, lysine, methionine, phenylalanine, threonine, tryptophan, and valine	Sui et al. (2021)
Amaranth	15–16	Globulin, albumin, prolamin, and glutelin	Source of lysine Limited content of Leucine	Zhu (2023)
Peanut	22–30	Globulin and albumin	Limited content of lysine, methionine, or threonine	Settaluri et al. (2012)
Walnut	18–24	Globulin, albumin, gliadin, and glutenin	Source of essential amino acids: arginine, glutamic acid, histidine, and tyrosine	Wen et al. (2023)

### Bioactive peptides

A characteristic considered in the value of proteins is bioactive peptides, composed of 2–20 amino acids linked by peptide bonds. Bioactive peptides are encrypted in the primary structure of plant proteins as inactive amino acid sequences, but they can be released by fermentation, food processing, and enzyme-catalyzed proteolysis in vitro or in the digestive tract after human consumption (Hartmann and Meisel, 2007). The importance of bioactive peptides has increased, particularly in the field of beneficial effects on health, generating an effect beyond normal and adequate nutrition (Zaky et al., 2022). Oat protein-derived bioactive peptides have been identified to exert distinct health-improvement properties, such as immunomodulatory, antifatigue, anti-thrombosis, anti-hypoxic, anti-hypertensive, hypocholesterolemic, and antioxidant effects (Rafique et al., 2022). Evidence shows that peptides from corn have antioxidant, anti-hypertensive, hepatoprotective, anti-inflammatory, anticancer, and dipeptidyl peptidase IV-inhibitory activities (Zhu et al., 2019). Also, the beneficial effects of peptides from soybeans include lipid-lowering (hypocholesterolemic and hypotriglyceridemic), anticancer, hypotensive, anti-inflammatory, and antioxidant effects in a variety of experimental models (Chatterjee et al., 2018). Peptides from amaranth show antioxidant, anti-atherosclerotic, hypocholesterolemic, anticoagulant, antiviral, anticancer, anti-inflammatory, dipeptidyl peptidase IV-inhibitory, and anti-hypertensive effects (Zhu, 2023). Peptides obtained from peanuts show antioxidant, antimicrobial, immunomodulatory, and anti-hypertensive effects (Shi et al., 2014). Walnuts contain peptides that show antioxidant, anti-hypertensive, and xanthine oxidase inhibitory activities (Li et al., 2020).

### Protein signaling pathways

The quality of the dietary protein has a fundamental impact on muscle protein synthesis, highlighting the importance of selecting appropriate protein sources. Amino acids, key components of proteins, play a crucial role in the body's homeostasis. They act not only as building blocks for the synthesis of proteins and peptide hormone, but also as an alternative energy source (Battu et al., 2017; Wu, 2014). This multifunctionality underscores their importance in maintaining health and overall well-being.

Protein synthesis represents a highly energetic biological process that takes place within the cell. The cells have undergone evolutionary changes to be able to identify and respond to the availability or scarcity of essential nutrients. In the case of eukaryotic cells, the detection of sufficient amino acids is accomplished through the mechanistic target of rapamycin (mTOR), whereas the insufficiency of a single essential amino acid or non-essential amino acid is manifested by an increase in uncharged cognate transfer RNA (tRNA), which is identified by the general control non repressible 2 (GCN2) (Battu et al., 2017).

## Structure of mTORC1

The mTOR is a Ser/Thr protein kinase found in two complexes, mTORC1 and mTORC2 (Fernandes and Demetriades, 2021). mTORC1 is composed of three core components: mTOR, the catalytic subunit of the complex; regulatory-associated protein of mTOR (Raptor), which facilitates substrate binding to mTOR (Yoon and Choi, 2016); and mammalian lethal with SEC13 protein 8 (mLST8, also known as GbL), which stabilizes the union between Raptor and mTOR and stimulates the mTOR kinase activity (Melick and Jewell, 2020; Zhang et al., 2021). In addition to the core components, mTORC1 has two inhibitory subunits: the 40 kDa Pro-rich AKT substrate (PRAS40, also known as AKT1S1) and the DEP domain-containing mTOR-interacting protein (DEPTOR) (Saxton and Sabatini, 2017).

Different stimuli, such as intracellular energy status, growth factors, oxygen levels, stress factors, and the availability of amino acids, regulate mTORC1 (Li and Yan, 2019). There is a reduction of mTORC1 activity when decrease the concentration of growth factors, amino acids, or energy status (AMP increase). PRAS40 and DEPTOR bind to the complex, where they promote the inhibition of the complex. PRAS40 regulates the kinase activity of mTORC1 by acting as a direct inhibitor of substrate binding (Han et al., 2022).

### mTORC1 signaling pathway

The activation of mTORC1 decreases catabolic pathways and enhances lipid, nucleotide, and protein synthesis (Liu and Sabatini, 2020). As previously stated, nutritional cues have a significant impact on mTORC1. For instance, insulin and/or growth factors regulate the mTORC1 signaling pathway through the phosphoinositide 3-kinase (PI3K)/Akt pathway. The serine/threonine kinase Akt turns on mTORC1 by blocking the tuberous sclerosis complex (TSC), which is made up of TSC1 (hamartin), TSC2 (tuberin), and TBC1D7 (Tre2-Bub2-Cdc16 Domain Family Member 7) (Condon and Sabatini, 2019). Several studies have demonstrated that the TSC complex is a key regulator of mTORC1, and depending on nutrient supply, it can upregulate or downregulate its activity (Rehbein et al., 2021; Zheng et al., 2014). TSC acts as a GTPase-activating protein (GAP) that regulates the activity of Rheb (Ras Homolog) (Liu and Sabatini, 2020). When the activity of TSC is inhibited, Rheb is in its active form (Rheb-GTP), allowing the activation of mTORC1 (Yang et al., 2017).

The amino acid availability activates the mTORC1 pathway through an independent mechanism of the PI3K/Akt pathway. The amino acids regulate the mTORC1 pathway through Rag GTPases, which are members of the GTP-related Ras proteins. Rag GTPases consist of four small GTPases (A–D), which exist as heterodimers between Rag A or B and Rag C or D and localize to the lysosomal membrane (Lama-Sherpa et al., 2023). The amino acids promote the binding of Rag A/B with GTP and later interact with mTORC1 through Raptor, favoring the translocation of mTORC1 to the lysosomal membrane. Rags GTPases are anchored to the lysosomal membrane by a complex called Ragulator and are responsible for loading Rag A/B with GTP due to their GEF (guanine nucleotide exchange factor) function. In turn, regulator function is controlled by lysosomal v-ATPase, which undergoes conformational changes when amino acids accumulate in the lysosomal inner membrane (Condon and Sabatini, 2019; Yao et al., 2017; Zhang et al., 2021).

### mTORC1 target protein

**Fig. 1:**
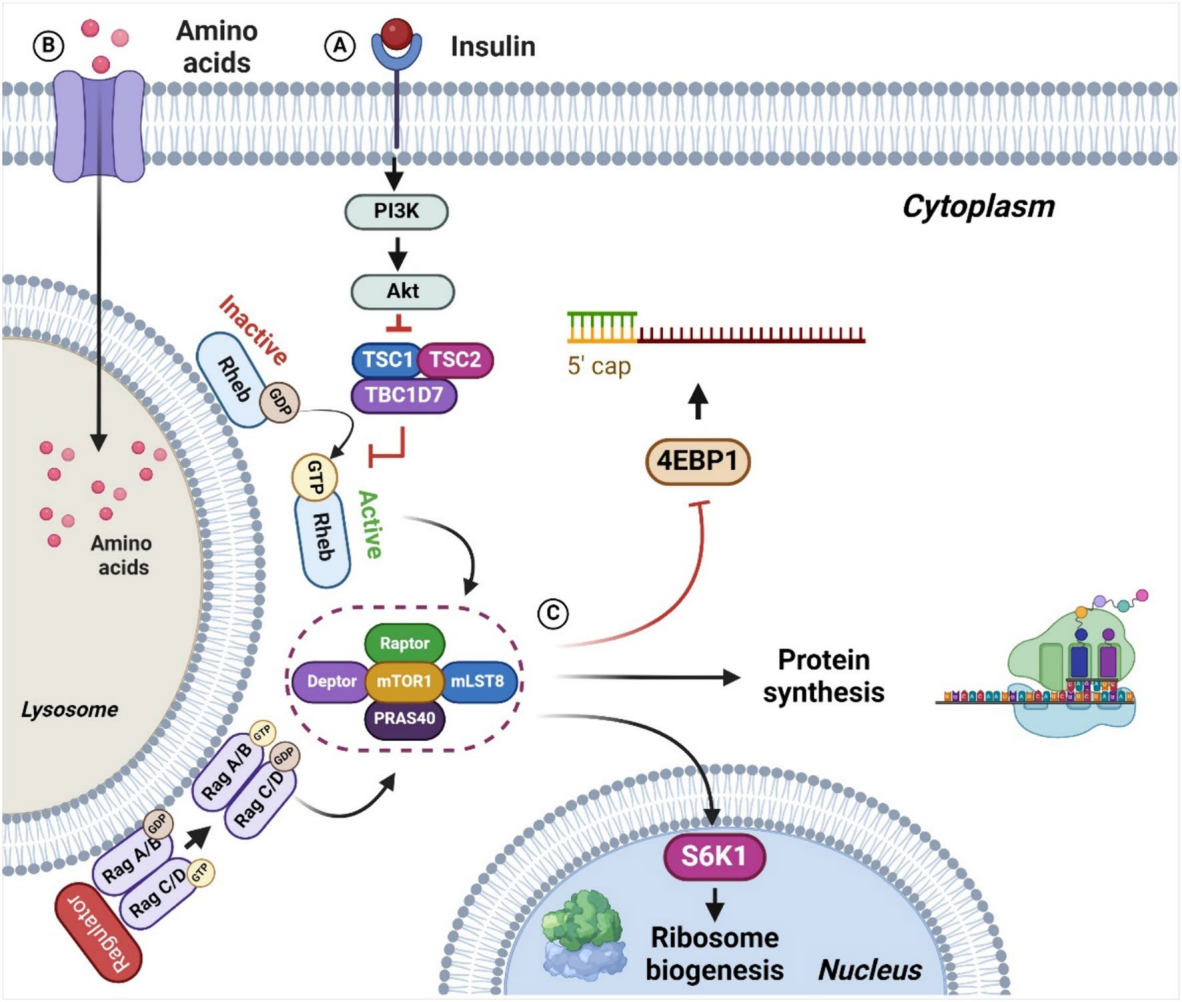
mTOR signaling pathway. (**A**) Insulin signaling, insulin activates the PI3K/Akt pathway (Phosphoinositide 3-kinase, serine/threonine kinase) which inhibit the complex TSC (tuberous sclerosis complex (TSC), composed of TSC1 (hamartin), TSC2 (tuberin), and TBC1D7 (Tre2-Bub2-Cdc16 Domain Family Member 7) allowing the formation of the Rheb-GTP (Ras Homolog). (**B**) Amino acid signaling, amino acids activate Rag GTPases (GTP-related Ras proteins), which recruit mTORC1 (mammalian target of rapamycin) to the lysosome surface where it interacts with Rheb-GTP. (**C**) The activation of mTORC1 by Rheb-GTP, stimulates the activity of S6K1 (ribosomal protein S6 kinase) that promotes ribosomes biogenesis and phosphorilates 4EBP1 (initiation factor 4E-binding protein 1) allowing the 5ˊ cap translation initiation, thus overall promoting protein translation

mTORC1 stimulates growth, cell proliferation, and macromolecule synthesis. It inhibits autophagy through the regulation of components of the translational machinery such as initiation factor 4E-binding protein 1 (4EBP1), ribosomal protein S6 kinase (S6K1), and elongation factor 2 (Beugnet et al., 2003). 4EBP1 binds to eukaryotic initiation factor 4E (4eIF4E), preventing 5ˊ cap translation initiation. The assembly of the translation initiation complex is made possible by the release of 4eIF4E caused by the phosphorylation of 4EBP1 by mTORC1 (Wu et al., 2017). In contrast to its suppression of 4E-BP1, mTORC1 activates S6K1 through phosphorylation. By increasing RNA polymerase I activity, active S6K1 promotes transcription of ribosomal RNAs (Fernandes and Demetriades, 2021). Therefore, the activation of mTORC1 stimulates the assembly of the translation machinery in response to nutritional status (Fig. 1).

## Mechanism of action of GCN2

**Fig. 2:**
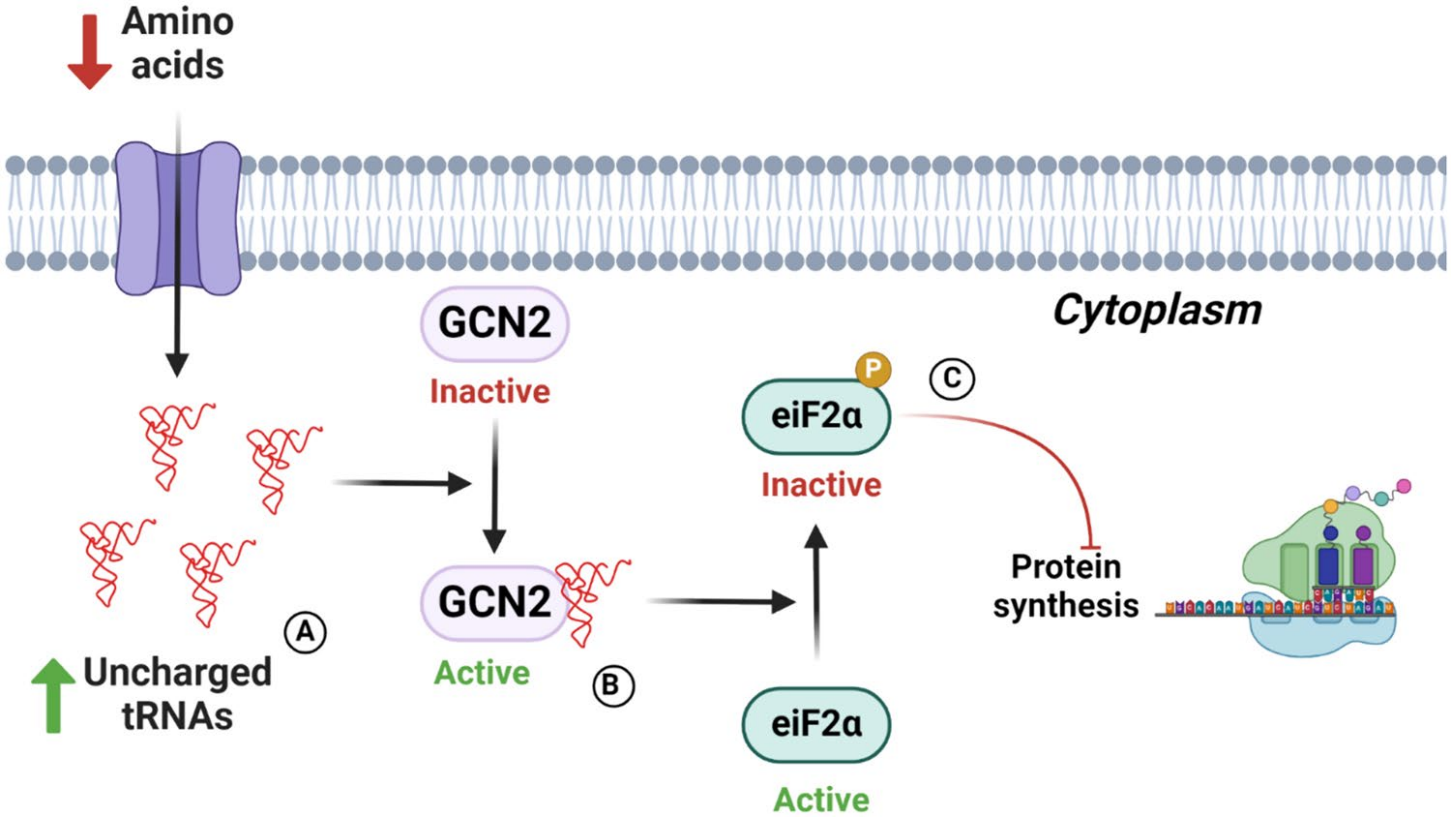
GCN2 molecular signaling pathway. (**A**) Amino acid deficiency increases uncharged tRNAs. (**B**) The increase of uncharged tRNAs is sensed by GCN2 (general control non repressible). **C** The activation of GCN2 by uncharged tRNAS, stimulates the phosphorylation of eIF2a (initiation factor α subunit of eukaryotic initiation factor 2) inhibiting the translation initiation, thus overall decreasing protein translation

The GCN2 is a serine/threonine kinase that regulates protein synthesis initiation (Battu et al., 2017). During amino acid scarcity, there is an increase in the concentration of uncharged tRNAs which are sensed by GCN2. The activation of GCN2 by this cue causes the phosphorylation of the translation initiation factor α subunit of eukaryotic initiation factor 2 (eIF2α) thus inhibiting protein synthesis (Li et al., 2022) (Fig. 2).

**Fig. 3:**
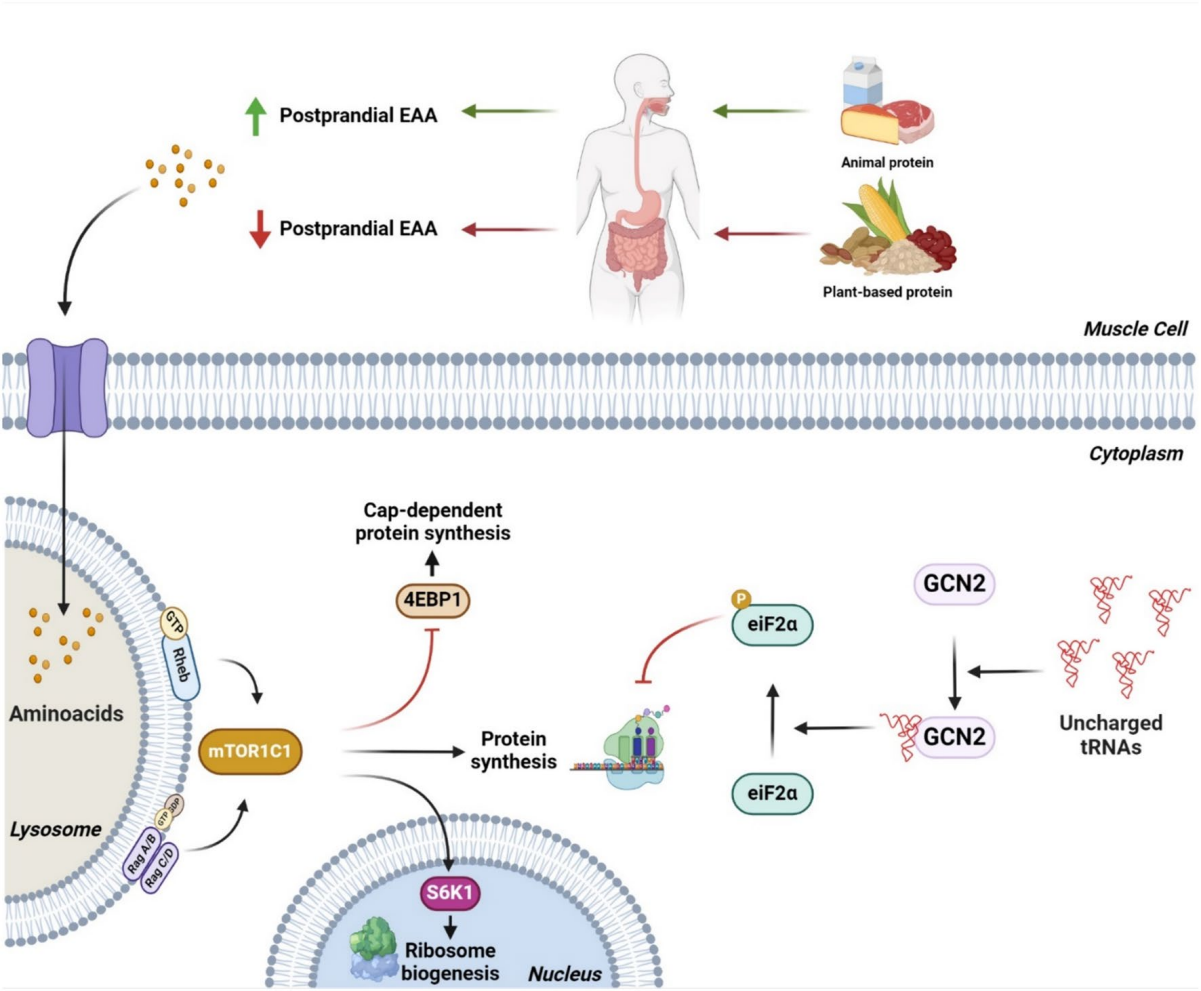
Overview of muscle protein synthesis. Animal protein (green arrows) consumption increases the serum concentration of EAA (essential amino acids) stimulating muscle protein synthesis though the activation of the mTOR signaling pathway. Plant based protein (red arrows) consumption causes a lower postprandial EAA (essential amino acids) concentration that downregulate the activation of mTOR and stimulate the activation of GCN2 causing a decrease in muscle protein synthesis

### Plant-based proteins and muscle proteins synthesis

By 2050, it is predicted that there will be 10 billion people on the planet, which raises concerns about food security because there will not be enough animal sources of protein to meet the demand (D'Hulst et al., 2021). Recent research has assessed the usage of plant-based protein to encourage more sustainable food systems. However, there are concerns regarding substituting plant protein for animal protein, primarily due to the lower protein quality of plant-based protein compared to animal protein, which could result in a reduced rate of muscle protein synthesis (Nichele et al., 2022). As previously stated, the amino acid profile has an important role in the activation of the mTORC1 signaling pathway, and it has been well established that after eating animal protein, the serum concentration of EAA rapidly increases, stimulating muscle protein synthesis (Kitada et al., 2019). On the other hand, plant-based proteins contain molecules such as phenolic compounds and anti-nutritional factors that might affect protein digestion, causing a lower postprandial EAA concentration (Bos et al., 2003). Current research focuses on methods that raise the nutritional value of plant-based proteins to equal the EAA content of sources of protein obtained from animals (D'Hulst et al., 2021) (Fig. 3).

### Clinical evidence of plant-based protein

Currently, there is an increase in interest in plant-based diets for various reasons, including consumer personal health reasons, caring for the environment, concerns for animals, religious beliefs, money saving, weight loss, and taste preferences (Rosenfeld and Burrow, 2017). The aim of plant-based diets is to replace the consumption of proteins of animal origin with the consumption of proteins of vegetable origin (Elliott et al., 2022). However, one of the most interesting questions is: What is the effect of the consumption of plant-based proteins on health? Starting from the point that dietary proteins are so important for human nutrition and their principal function is tissue-building, other functions are related to body composition and regulating metabolic pathways such as satiety and immune system activity (Jahan-Mihan et al., 2011).

This pattern is characterized by a greater intake of certain major food categories that are based on plant-based protein sources such as legumes, cereals (whole grains), seeds, and nuts, as well as the consumption of fruits, vegetables, and tubers (Visioli et al., 2000). Plant-based diet patterns can be dietary patterns in which foods of animal origin are totally or mostly excluded, so ovolactovegetarian, flexitarian, pescetarian, and vegan diets are considered within this pattern (Hargreaves et al., 2023). There is evidence that well-planned plant-based protein patterns can provide the same quality of protein and similar amounts of nitrogen as animal protein or a mixed diet and can also provide adequate nutrient intake (Ferrari et al., 2022). Also, some studies demonstrate, particularly in subjects with obesity and insulin resistance, that either animal or vegetable high-protein hypocaloric diets improve insulin sensitivity by 60–90% (Gonzalez-Salazar et al., 2021).

Table 3 shows the available clinical evidence of the consumption of vegetable protein in different outcomes. In cereals we have options such as oats and corn, it is based on the cereal grains are the main dietary source of energy, carbohydrates, and plant proteins worldwide, and they are better known for their contribution of dietary fiber to food, and important protein contribution. The protein content of cereal grains varies between 7–8% of dry matter (Poutanen et al., 2022). Oats have higher concentrations of EAA, specifically concentrations of lysine, which is the crucial limiting amino acid in wheat and other grains (Chu, 2013). On the other hand, corn is the third most consumed cereal as human food, after rice and wheat. The three big global staple cereals, wheat, rice, and maize, comprise a major component of the human diet, accounting for an estimated 42% of the world’s food calories and 37% of protein intake, and food consumption of maize grain contributes 5% of the total human dietary calories and proteins globally (Erenstein et al., 2022).

**Table 3:** Clinical evidence of plant-based protein

Source	Study type	Population	Intervention	Principal results	Conclusion	References
Cereals						
Oats	Randomized controlled trial	16 male athletes with a mean age of 19 ± 1.1 years	Consumption of oat protein (25 g of protein) before an exhaustive downhill running test and then during the 4 days after and compared vs. the placebo group (maltodextrin)	**Oat protein group:** ↓ AUC of the concentration of CRP from pre-exercise to 96 h post-exercise, (15.48 ± 2.51 vs. 11.90 ± 3.44, *p* = 0.032) ↓ IL-6 concentrations were 29.2% lower post-exercise ↓ VAS value for leg muscle pain after downhill running (AUC: 30.29 ± 1.65 vs. AUC: 18.29 ± 2.95, *p* < 0.01)	These findings demonstrated that oat protein supplementation has the potential to alleviate the negative effects of eccentric exercise in untrained young men	Xia et al. (2018)
Corn	Randomized, double-blind, parallel-group study	36 healthy young men (26 ± 4 years)	30 g of milk protein (MILK) *vs* 30 g of corn protein (CORN) *vs* mixture of 15 g of corn protein plus 15 g of milk protein (CORN + MILK)	MILK: ↑ plasma EAA (iAUC: 151 ± 31 vs. 77 ± 19 mmol/L/300 min, *p* < 0.001) MILK: ↑ plasma EAA iAUC vs CORN + MILK (151 ± 31 vs. 126 ± 24 mmol/L/300 min, p = 0.036) MILK and CORN: ↑ myofibrillar protein synthesis rates (*p* < 0.001) with no differences between MILK and CORN (from 0.014 ± 0.014 to 0.053 ± 0.013 and from 0.017 ± 0.011 to 0.052 ± 0.013%/h, *p* = 0.661)	Postprandial muscle protein synthesis rates after ingestion of MILK protein do not differ from rates observed after ingestion of CORN protein or a mixture of MILK and CORN protein	Pinckaers et al. (2020)
Legumes						
Soy	Systematic review	Older adults	Supplementation with isolated soy protein	↓ Serum TNF-α levels (MD: − 0.16, CI 95% [− 0.26, − 0.05], I^2^ = 68%)	Decrease of TNF-α could be associated with the prevention and treatment of sarcopenia	Prokopidis et al. (2023)
	Cross-sectional study	29,525 adults	Consuming soy food more than 4 times/week *vs*. soy food < 1, 1 and 2–3 times/week	**High soy food consumption:** ↑ HGS 36.6 [35.1, 38.0] kg, *p* < 0.001 *Analysis was adjusted for age, sex and BMI, smoking status, alcohol drinking status, educational level, occupation, monthly family income, physical activity, hypertension, hyperlipidemia, total energy intake, and dietary pattern	Soy food that contains high levels of soy protein or isoflavones can help to stimulate muscle protein synthesis and ameliorate muscle strength decline. The consumption of soy foods may have beneficial effects on muscle health	Wu et al. (2023)
	Randomized clinical trial	Patients with type 2 diabetes	Combination of high-fiber, polyphenol-rich, and vegetable-protein functional foods, the plant-based protein included in the intervention was 30 g of soy protein *vs.* placebo	**Combination of functional foods:** ↑ alpha diversity of fecal microbiota Relative abundance of bacteria in fecal microbiota ↓ *Prevotella copri* ↑ *Faecalibacterium prausnitzii* ↑ *Akkermansia muciniphila* ↓ Glucose, total and LDL cholesterol, free fatty acids, HbA1c, triglycerides, and CRP in plasma	These patterns that contain plant-based proteins provide benefits for the composition of the fecal microbiota and may offer potential therapies by improving glycemic control, dyslipidemia, and inflammation	Medina-Vera et al. (2019)
Beans	Randomized crossover 2 × 2 trial	Adults with BMI 25–29.9 kg/m^2^ and blood lipid alteration	32 g of the common bean baked snack daily (8.6 ± 0.1 g of protein and 5.2 ± 0.2 of total dietary fiber) for four weeks	↓ Blood levels of ApoB-100 (77.2 ± 22.1 to 56.6 ± 12.7 mg/dL)	ApoB-100 has a central role in the development of atherosclerosis, so its decrease after the intervention seems to be an important premise	Escobedo et al. (2021)
Pseudo-cereals						
Amaranth	Randomized, double-blind trial	6-Month-old infants (n = 499)	Nine monthly servings of: -WinFood Classic (WFC) (71% sprouted amaranth, 10.4% corn, 3% small fish, and 10% edible termites) -WinFood Lite (WFL) (82.5% sprouted amaranth 10.2% corn and multi-micronutrient premix) -Fortified Corn Soy Blend plus (CSB +)	**In comparison with CSB + **change from 6 to 15 months **FFM**: no significant differences were observed with the WFC group (0.0 kg, CI95% [− 0.30, 0.29]) and with the WFL group (0.03 kg, CI95% [− 0.25, 0.32]) **Length:** no significant differences were observed with the WFC group (-0.1 cm, CI95% [− 0.30, 0.2]) or with the WFL group (-0.1 cm, CI95% [− 0.30, 0.1]) **Weight:** no significant differences were observed for the WFC (-0.3 kg, CI95% [− 0.9, 0.4]) and WFL groups (-0.3 kg, CI95% [− 0.9, 0.3])	WinFoods (which was mostly sprouted amaranth) had the same effect on FFM, length, and weight gain as CSB +	Konyole et al. (2019)
Nuts						
Peanut	Randomized control trial	Older and untrained individuals 58.6 ± 8.0 years BMI 28.7 ± 5.8 kg/m^2^	Peanut protein supplement once per day (75 total g of powder providing 30 g protein, > 9.2 g essential amino acids, ~ 315 kcal)	↑ muscle *Vastus Lateralis* thickness ↑ flexion torque	Higher protein, defatted peanut powder supplement combined with a resistance training program positively affects select markers of muscle hypertrophy and strength in the study population	Lamb et al. (2022)
Walnut	Randomized crossover trial	90 healthy adults (54.3 years old)	Daily supplementation of nuts (12% of their daily energy intake) in the usual diet *vs*. control diet for 6 months	**Walnut supplementation:** ↑ plant-based protein, total fat, total PUFA, and total dietary fiber (*p* < 0.05) ↑ Mineral levels including calcium, phosphorous, magnesium, and zinc (*p* < 0.05)	Nutrient and food displacement may be mechanisms to explain the favorable association between nut consumption and an improved diet, this may contribute to the prevention of chronic diseases	Natto et al. (2022)

*AUC* area under the curve, *IL-6* Interleukin-6, *VAS* visual analog scale, *EAA* essential amino acid, *iAUC* incremental area under the curve, *HGS* handgrip strength, *TNF-α* tumor necrosis factor-alpha, *LDL* low-density lipoprotein, *CRP* C-reactive protein, *BMI* body mass index, *ApoB-100* apolipoprotein B-100, *FFM* Fat-Free Mass, *PUFA* Polyunsaturated fatty acid

↓ decrease, ↑ increase

Within the group of legumes, we have two foods that are very popular and are mostly consumed in diet patterns based on plant-based proteins: soy and beans. Soy protein is one of the major sources of plant-based protein for human consumption, and its consumption has been associated with beneficial effects on health and common bean (*Phaseolus vulgaris L*.) has some bioactive compounds that impact health. These bioactive compounds are proteins, dietary fiber, linoleic and oleic acids, polyphenols, saponins, and phytosterols (Celmeli et al., 2018). Although protein content is very variable depending on the bean variety, approximately half a cup of cooked beans has 25 g of protein (Ganesan and Xu, 2017), which can be considered a good source of protein in the diet. On the side of the pseudo-cereal, we have amaranth with an important contribution of protein to the diet with an average protein content of 17.9% (Orona-Tamayo and Paredes-López, 2017).

## Food security, food safety, and public health

Plant-based protein diets are emerging as promising diets to prevent and treat diseases as well as an option for the sustainability of the planet. Therefore, their use is expanding, which implies greater production and marketing of so-called food supplements based on vegetable proteins. However, it is necessary to review food safety to guarantee the well-being and health of the consumer (Ionel, 2018). European legislation can be defined as a sum of regulatory acts of a strictly legislative and/or administrative nature, which regulate the veterinary health area and food safety, which is complex and constantly evolving, these regulations must focus on production conditions and marketing of supplements and foods that provide plant-based protein, ranging from the production, circulation and marketing of these products; and great care must be taken in regulating that the products are within the regulatory limits of residues of antibiotics and antiparasitic and/or biocidal substances and other products in the production of plant-based protein products, because all this directly or indirectly influences food safety, as well as consumer health (Ionel, 2018). Given that many food safety issues are in continuous evolution, such as these diets and/or plant-based protein products, the regulation must also adapt to this evolution, with the aim of providing an immediate solution in a very short period. Only to important animal health issues, but also public health issues, including those that endanger or could endanger the health of the consumer (Ionel, 2016). Although diets based on plant protein seem to be promising in the future, the guidelines for regulating food safety must be monitored, an area in which much remains to be done, but which should not be lost sight of, to the public health of consumers.

## Technologies for plant-based protein extraction

In recent years, the modern consumer has come to believe that nutrition can play an essential role in disease prevention and health promotion. This fact has increased the demand for high-quality protein products for the daily diet, and it has been suggested that substituting meat with plant-based foods has some health-promoting advantages (Feher et al., 2020). Moreover, the increasing popularity of plant-based diets and current trends in reducing meat consumption have stimulated a growing research interest in exploring novel plant protein sources and developing suitable cost-effective and eco-friendly technologies to produce plant protein-rich ingredients with enhanced functionality to be used in the development of new and better protein-based food products (Franca-Oliveira et al., 2021).

Among factors that hinder the extraction and purification of plant-derived proteins are intrinsic structural characteristics of plant proteins and their complexation with other minor components from plant matrices (e.g., phytates, tannins, fibers, non-starch polysaccharides, and other antinutritional molecules) (Sá et al., 2019). Different technologies are used for protein extraction from diverse plant sources. These technological methods are generally classified into wet and dry methods. In the protein industry, wet extraction is the most used. This method is based on the utilization of acidic, alkaline, salt, or alcohol solutions for protein extraction (Amin et al., 2022).

Generally, protein solubility increases as the pH of the extraction solution increases due to the ionization of acidic and neutral amino acids at high pH (Kumar et al., 2021). Therefore, protein alkaline extraction is the most frequently used method because most proteins from plant sources achieve good solubility under these conditions (Franca-Oliveira et al., 2021).

All these methods, considered conventional extraction techniques, have some disadvantages, such as being energy-intensive, time-consuming, and not eco-friendly due to using alkalis, acids, and organic solvents (Kumar et al., 2021). The most prominent alternatives to conventional protein extraction techniques are emerging technologies assisted by ultrasound, enzymes, microwaves, high hydrostatic pressure, or pulsed electric fields. These methods could potentially increase the protein extraction yield while reducing chemicals and water consumption (Franca-Oliveira et al., 2021; Sá et al., 2019). The pros and cons of different protein extraction technologies are summarized in Table 4.

**Table 4:** Pros and cons of technologies for plant-based protein extraction

Method	Feature	Pros	Cons	References
Conventional extraction	The most common methods involve the use of alkaline, acidic, salt, or alcohol solutions	↑ Can be used on a broad range of plant matrices	↑ Energy-intensive and time-consuming ↓ Not eco-friendly ↓ Protein extraction yield	Kumar et al. (2021)
Ultrasound-assisted extraction	Uses ultrasonic waves that pass through the liquid extraction medium, generating the expansion and collapse of bubbles causing cavitation. The ultrasound cavitation facilitates the solid–liquid interaction and leads to cell wall rupture, particle size reduction, and mass transfer across cell membranes	↓Extraction time ↓ Extraction temperature ↓ Solvent consumption ↑ Heat-sensitive molecules preservation ↑ Protein extraction yield	↑ Protein structural modifications ↑ Difficult to scaling up	Ampofo and Ngadi (2022) and Bernardi et al. (2021)
Enzyme-assisted extraction	Based on the disruption of the structural integrity of the cell walls through the degradation of their major components by the action of non-starch polysaccharide degrading enzymes (e.g.,cellulose, hemicellulose, and/or pectinase)	↓ Negative impact on the environment ↑ Protein extraction yield	↑ Energy consumption ↑ Processing time ↑ Operating costs ↑ Difficult to scaling up	Pojic et al. (2018) and Puri et al. (2012)
Microwave-assisted extraction	Uses non-ionizing electromagnetic waves (300 MHz-300 GHz) that overheat water molecules in the cells and consequently generate high pressure on the cells’ walls, increasing their porosity and favoring solvent penetration and protein extraction	↓ Extraction time ↓ Solvent consumption ↑ Protein extraction yield	↑ Thermal energy ↑ Degradation of heat-sensitive proteins and bioactive compounds	Franca-Oliveira et al. (2021) and Ochoa-Rivas et al. (2017)
High hydrostatic pressure-assisted extraction	Non-thermal technology based on the application of isostatic pressure (100 to 1000 MPa) that is transmitted instantaneously and uniformly through the extraction medium. The applied pressure induces cell deformation and cell wall damage, allowing the solvent to penetrate the cell and increase the mass transfer	↓ Solvent consumption ↓ Extraction time ↑ Protein extraction yield ↑ Heat-sensitive molecules preservation ↓ Protein allergenicity	↑ Protein structural modifications ↑ Operating costs	Dehnad et al. (2023), Li et al. (2016) and Sá et al. (2019)
Pulsed electric field-assisted extraction	Consists of subjecting the extraction matrix to electric pulses with high-intensity electric fields (0.1–80 kV/cm) during short periods (from microseconds to milliseconds). Exposure to high electric fields induces the formation of pores within the cellular structures, increasing the permeability of the cell membrane	↓ Extraction time ↓ Energy consumption ↑ Protein techno-functionality	↑ Operating costs ↑ Requirement of optimized processing conditions to improve protein extraction yield	Golberg et al. (2016), Kamboj et al. (2022), Melchior et al. (2020) and Rodrigues et al. (2020)

↓ decrease, ↑ increase

Regardless of the wet extraction method used, the extracted protein needs to be concentrated or isolated to be transformed into a plant-based ingredient or product. Most of the commercially available protein concentrates are obtained by the conventional alkali extraction method, followed by iso-electric precipitation at acid pH. Acidic conditions are commonly reached by hydrochloric acid addition, which can lead to racemization and the loss of some amino acids, causing impaired digestibility. Moreover, the use of strong acids induces severe protein denaturation and brown substances (Yadav et al., 2022), besides not being an eco-friendly technique (Kumar et al., 2021).

To avoid the drawbacks associated with the conventional process, some other technologies have been developed for protein recovery and concentration/isolation.

Membrane separation techniques, in particular ultrafiltration, are an improved method used at laboratory and industrial scales to concentrate protein extracts (1–1000 kDa) (Vijayasanthi et al., 2020). Protein concentrates obtained by this method have shown enhanced functional properties compared to those obtained by acid precipitation. Due to its mild operating conditions and low energy requirement, ultrafiltration seems to be a good alternative to producing protein isolates (John et al., 2021).
